# Cotyledonoid Dissecting Leiomyoma: A Rare Case With Rapid Regrowth After Fertility-Sparing Surgery and Hormonal Therapy

**DOI:** 10.7759/cureus.97793

**Published:** 2025-11-25

**Authors:** Yuriko Nakamura, Shizuka Yamada, Toshimichi Onuma, Makoto Orisaka, Yoshio Yoshida

**Affiliations:** 1 Obstetrics and Gynecology, University of Fukui, Fukui, JPN; 2 Obstetrics and Gynecology, Fukui Aiiku Hospital, Fukui, JPN

**Keywords:** 18f-fluoroestradiol pet, cotyledonoid dissecting leiomyoma, fertility preservation, gonadotropin-releasing hormone antagonist, hormonal therapy, recurrence, uterine leiomyoma

## Abstract

Cotyledonoid dissecting leiomyoma (CDL) is a rare benign variant of uterine leiomyoma. Its multinodular, placental-like morphology often mimics malignant tumors, leading to overtreatment.

We report a case of a 34-year-old nulliparous Japanese woman who presented with abdominal distension and urinary frequency, and was subsequently diagnosed with CDL. MRI revealed a 30 cm heterogeneous uterine mass. She desired to preserve fertility, so myomectomy was planned. However, because of the tumor’s characteristics, complete resection was difficult and partial resection of the tumor was performed due to intraoperative bladder injury, leaving an 8 cm residual tumor. Histopathology confirmed CDL. Despite postoperative gonadotropin-releasing hormone (GnRH) antagonist therapy, the tumor enlarged to 13 cm over nine months. ^18^F-fluoroestradiol (^18^F-FES) PET showed no tracer uptake. At the time of presentation to our hospital, she no longer desired fertility preservation. Therefore, she underwent a total hysterectomy with right salpingectomy as the second surgery, and histology confirmed recurrent CDL. She remains disease-free two years postoperatively.

CDL is a histologically benign tumor that can exhibit aggressive behavior when incompletely excised. Hormonal therapy appears to have limited efficacy, possibly due to impaired or functionally inactive hormone receptors. Complete surgical resection remains essential to prevent recurrence, whereas fertility-sparing surgery should be considered only when complete removal is technically achievable. Greater awareness of this rare but deceptive entity may help clinicians avoid unnecessary radical procedures and optimize outcomes for affected women.

## Introduction

Uterine leiomyomas, commonly known as fibroids, are the most prevalent benign tumors of the uterus. They present in a wide variety of histological forms. Among these, cotyledonoid dissecting leiomyoma (CDL) is an exceptionally rare subtype. First described by Roth in 1996, CDL presents as an exophytic, multinodular, grape-like or placental cotyledon-shaped mass arising from the uterine corpus and extending into the pelvic cavity or broad ligament [1]. Owing to its grossly lobulated configuration and seemingly infiltrative growth pattern, CDL is often mistaken for a malignant neoplasm such as leiomyosarcoma, leading in many cases to unnecessary radical surgery, including hysterectomy with bilateral salpingo-oophorectomy [2].

Distinguishing CDL from malignant uterine tumors is therefore critical. Microscopically, CDL can be distinguished from leiomyosarcoma by the absence of cytologic atypia, necrosis, or significant mitotic activity features. Although histologically benign, its atypical macroscopic appearance closely mimics aggressive disease. This diagnostic uncertainty poses a particular challenge in women of reproductive age, where the need for fertility preservation must be carefully balanced against oncologic safety. Because CDL remains exceedingly uncommon - with fewer than 120 cases indexed in PubMed to date - there are no standardized management guidelines, and most clinical decisions rely on individual case experience.

We describe the case of a 34-year-old woman with CDL who underwent fertility-sparing surgery complicated by subsequent tumor regrowth. This report underscores the diagnostic pitfalls and therapeutic challenges of CDL and reviews the relevant literature, emphasizing fertility preservation strategies, recurrence risk, and the potential role of hormonal therapy.

## Case presentation

A 34-year-old Japanese woman, gravida 0, para 0, presented with progressive abdominal distension and urinary frequency. Her medical history was notable for depression managed with paroxetine hydrochloride hydrate, quetiapine fumarate, flunitrazepam, and trazodone hydrochloride.

**Figure 1:**
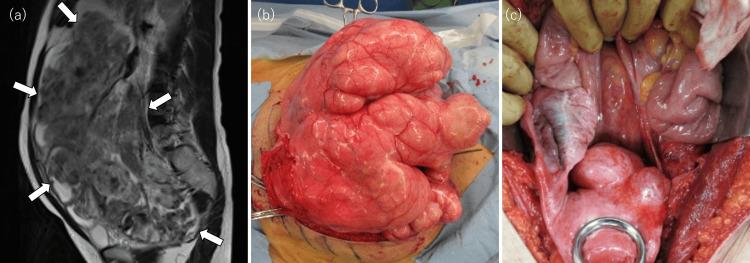
Findings from the previous hospital (a) MRI revealed a large pelvic mass (30cm) arising from the posterior wall of the uterus. (White arrows delineate the margins of the tumor.) (b) Intraoperative findings at the start of first surgery. The exophytic multinodular mass was arising from the uterine fundus. (c) Intraoperative findings at the end of first surgery. There was 8cm of remaining tumor arising from the posterior wall of uterus.

Magnetic resonance imaging (MRI) revealed a massive 30 cm pelvic mass arising from the posterior uterine wall (Figure 1A). On T2-weighted imaging, the tumor demonstrated a heterogeneous signal pattern compared with the myometrium, appearing subdivided into lobulated components by low-signal linear structures, within which areas of high-signal stroma were scattered. Laboratory tests, including tumor markers, showed values within the normal range (CEA 1.9 ng/mL, CA125 8.5 U/mL, LDH 139 U/L), and no other significant abnormalities were observed in the blood examination. Based on these findings, her previous physician diagnosed a large subserosal leiomyoma and initiated treatment with a gonadotropin-releasing hormone (GnRH) antagonist for two months, followed by exploratory laparotomy. Because the patient wished to preserve fertility, a myomectomy was planned.

At surgery, the mass appeared multinodular and exophytic, originating from the uterine fundus and compressing the bladder and left ureter (Figure 1B). Although no gross invasion of adjacent pelvic organs was observed, the tumor extended into the left broad ligament. The interface between the tumor and surrounding myometrium was poorly defined, making complete excision technically challenging. Intraoperatively, a bladder injury occurred and required immediate repair, limiting the extent of resection. Consequently, only partial removal of the tumor and left adnexa was achieved, leaving an approximately 8 cm residual mass (Figure 1C).

**Figure 2:**
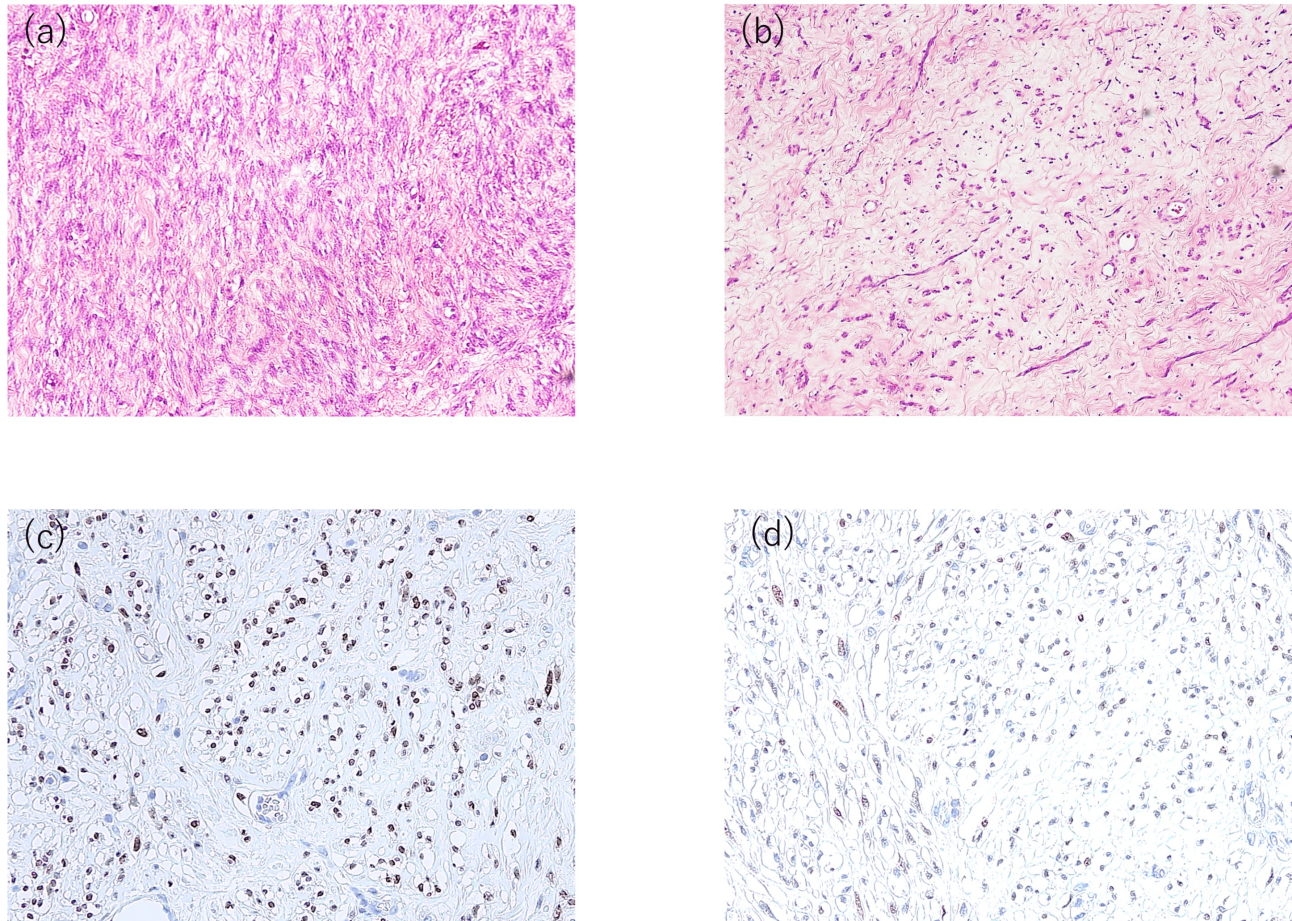
Pathological findings (a, b) Microscopic examination sections revealed the stiff area of fascicular spindle-shaped cells and the soft area of hydropic degeneration migrated to each other. (c) Immunohistochemical staining showed positive estrogen receptor expression. (d) Immunohistochemical staining showed positive progesterone receptor expression.

Grossly, the resected specimen was a lobulated soft-tissue mass measuring 26 × 26 × 10 cm and weighing 3.8 kg. The cut surface appeared grayish-white, glistening, and variably firm. Microscopically, the tumor consisted of alternating fascicles of spindle-shaped smooth muscle cells and areas of edematous myxoid stroma with prominent intercellular collagen deposition (Figure 2A, 2B). No cytologic atypia, tumor cell necrosis, or significant mitotic activity was identified (Ki-67 < 1%). Immunohistochemistry showed diffuse positivity for desmin, α-smooth muscle actin (αSMA), estrogen receptor (ER), and progesterone receptor (PR) (Figure 2C, 2D), confirming the diagnosis of CDL.

**Figure 3:**
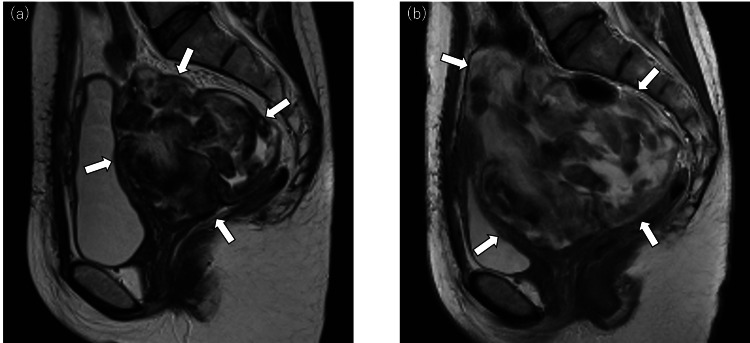
MRI findings at our hospital (white arrows delineate the margins of the tumor.) (a) MRI remained that the residual tumor grew to 10cm. (b) MRI revealed that the tumor grew to 13cm at the time of visiting our hospital.

Postoperatively, ultrasonography demonstrated an 8 × 6 cm residual mass, and GnRH antagonist therapy was resumed. Four months later, follow-up MRI revealed enlargement of the residual tumor to 10 cm (Figure 3A). The patient subsequently declined further fertility-preserving management and was referred to our institution nine months after the initial surgery. On presentation, MRI showed a now 13 cm mass with a more confluent appearance, lacking the distinct multinodular pattern previously observed (Figure 3B).

**Figure 4:**
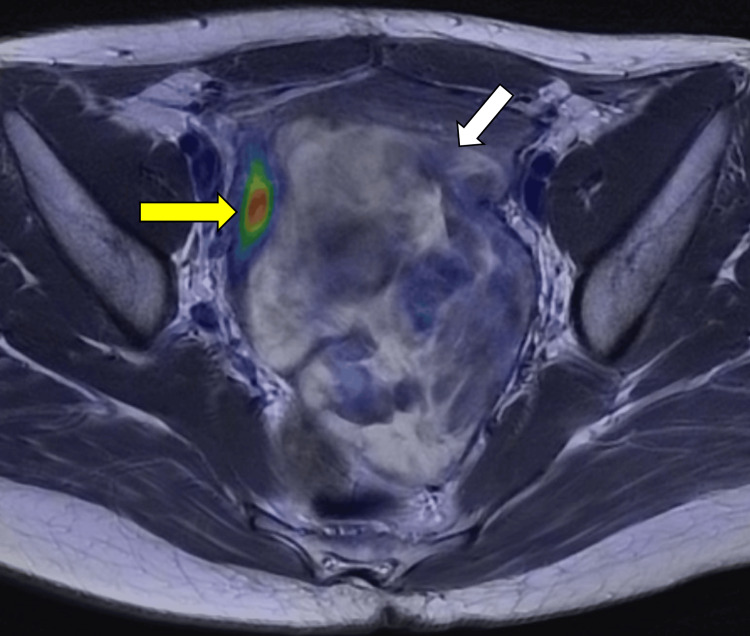
¹⁸F-fluoro-17β-estradiol (¹⁸F-FES) positron emission tomography (PET) findings ^18^F-FES PET MRI showed ^18^F-FES uptake was observed in the endometrium, and no evidence of accumulation in the tumor. (white arrow: tumor, yellow arrow: endometrium)

T2-weighted imaging demonstrated increased hyperintensity consistent with progressive edema. On 16α-^18^F-fluoro-17β-estradiol (^18^F-FES) positron emission tomography (PET), tracer uptake was evident in the endometrium but absent in the tumor (Figure 4).

**Figure 5:**
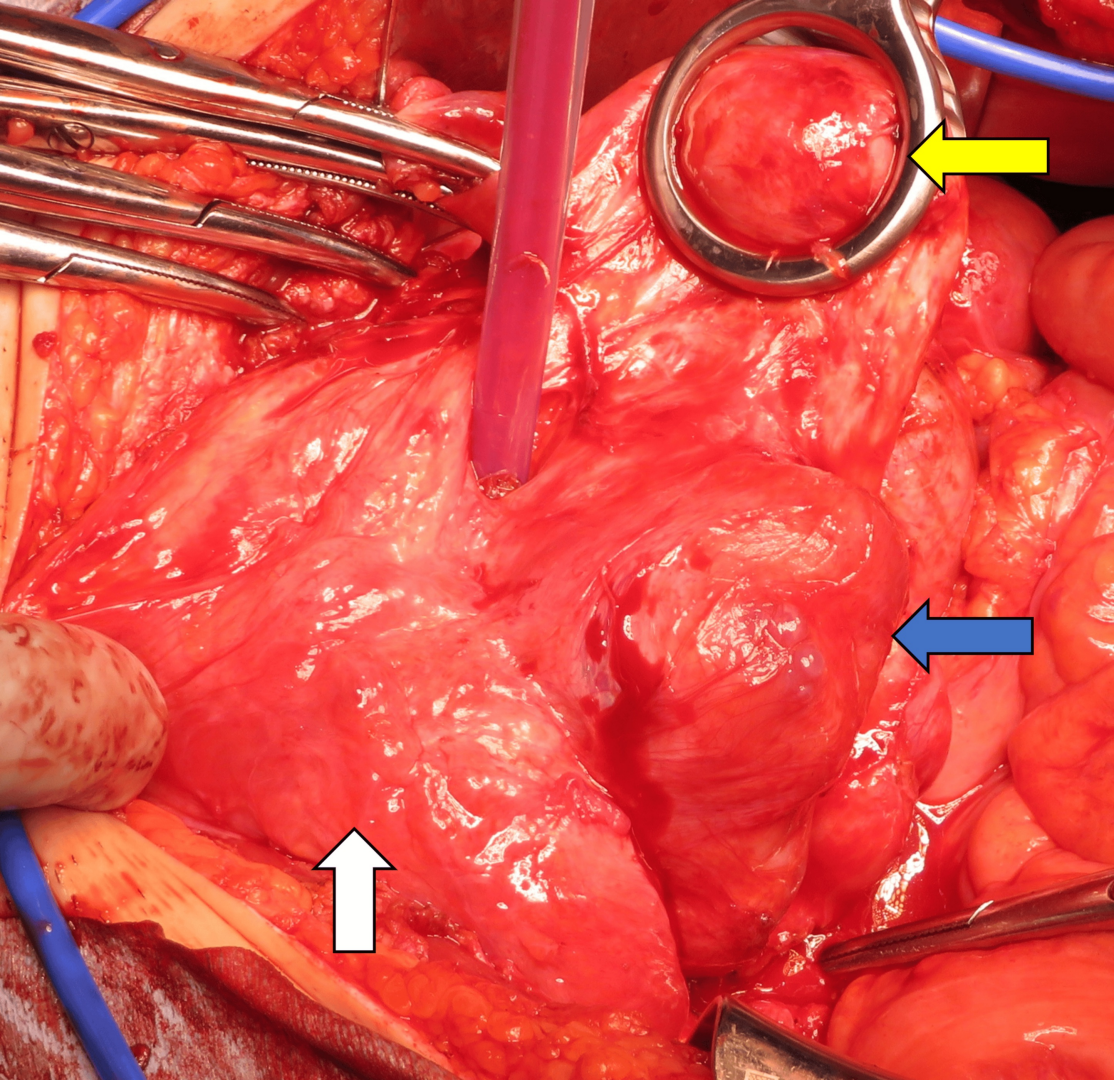
Intraoperative findings of second surgery Intraoperative findings in the middle of second surgery. It was difficult to distinguish the tumor and intestinal tract or bladder. (yellow arrow: uterus, white arrow: bladder, blue arrow: intestinal tract)

Given the tumor’s continued growth, a total hysterectomy with right salpingectomy was performed. Intraoperatively, the mass was extremely soft and adherent, making separation from the adjacent intestine difficult (Figure 5).

Dense adhesions related to the prior bladder injury required additional repair of the bladder serosa. Grossly, complete resection was achieved. Histopathological examination confirmed recurrent CDL with features identical to the previous specimen, and immunohistochemistry again demonstrated ER and PR positivity. The right ovary appeared normal and was preserved. At two-year follow-up, the patient remains disease-free, with no radiologic or clinical evidence of recurrence.

## Discussion

CDL is a rare histologic variant of uterine leiomyoma. Since Roth’s first description in 1996 [1], approximately 100-120 cases have been reported worldwide. CDL typically occurs in women of reproductive age, with a mean age of 44 years (range, 21-73) [2]. Clinical manifestations are nonspecific and primarily related to tumor size, including abdominal distension, pelvic pain, or abnormal uterine bleeding [3]. Grossly, CDL is characterized by multiple exophytic nodules resembling placental cotyledons that may extend into the broad ligament. Histologically, it is composed of interlacing fascicles of bland spindle cells interspersed with edematous or hyalinized stroma, lacking cytologic atypia, necrosis, or significant mitotic activity-features that distinguish it from leiomyosarcoma [1,4]. Immunohistochemical positivity for desmin, αSMA, ER, and PR confirms its smooth muscle origin.

Despite its benign histology, CDL frequently mimics malignancy intraoperatively. Its multinodular, grape-like appearance and indistinct margins often prompt suspicion of sarcoma, leading to radical surgery such as hysterectomy with bilateral salpingo-oophorectomy, even when frozen section analysis indicates benignity [5,6]. There are no established MRI characteristics of CDL; however, case reports have described that the tumor shows heterogeneous signal intensity on T2-weighted images compared with the myometrium, with patchy iso-intense nodules and thin high-signal stromal components. In addition, some reports have suggested that areas of high signal intensity within the mass on T2-weighted images reflect hydropic degeneration of the perinodular stroma in pathological findings [7]. These findings may be useful for preoperative imaging diagnosis, however these features cannot reliably exclude malignancy. In addition to such preoperative assessment, if MRI demonstrates features suggestive of CDL, the possibility of CDL should be considered. Once clinically suspected, management can be planned accordingly - for example, aiming for complete resection while recognizing the limited role of GnRH therapy. Rapid intraoperative pathological diagnosis may further help confirm the diagnosis and avoid overtreatment, which could be particularly beneficial for fertility preservation in young patients.

We summarized previously reported cases of CDL in women under 40 years of age (Table 1). The tumor size ranged widely from 4 to 41 cm (mean: 17.29 cm, median: 13 cm), and several reports described total hysterectomy even in young patients with relatively small tumors. In our case, the tumor removed during the first surgery measured 26 cm, which was the largest among the cases in which myomectomy had been attempted. This table illustrates that, even in younger women, many patients undergo total hysterectomy as the initial surgical approach [1,3-6,8-15].

**Table 1:** Summary of cases aged 40 years or younger Abbreviations: CDL: cotyledonoid dissecting leiomyoma, AUB: Abnormal uterine bleeding, ATH: abdominal total hysterectomy, BSO: Bilateral Salpingo-oopherectomy, BS: Bilateral Salpingectomy, LS: Left Salpingectomy, RSO: right salpingo-oopherectomy, OMT: Omentectomy, NA: not available, NED: No Evidence of Disease * In this case, there was residual tumor but no subsequent increase in size.

Author	Age	Symptom	Size (cm)	Rapid Intraoperative Pathologic Diagnosis	Surgery	Outcome and observation period (months)
Roth LM [1]	23	pelvic mass, AUB	25	-	resection of tumor	NED, 192
Kim MJ [4]	26	pelvic mass, AUB	5	benign smooth muscle	resection of tumor	NED, 24
Saeki H [8]	31	abdominal pain	13	leiomyoma	myomectomy	pregnancy after 1 year NED, 54
Sonmez FC [11]	38	abdominal pain	13	-	myomectomy	NED, 36
Tanaka H [9]	36	medical check up (no symptoms)	10	-	LSO + resection of tumor	NED, 26
Roth LM [10]	33	AUB	13	CDL	myomectomy	recurrent at 37y.o. and ATH+RSO
Brand AH [12]	24	pelvic mass	NA	benign smooth muscle	resection of tumor	NED 15＊
Jordan LB [13]	36	hypermenorrhea	13	-	subtotal hysterectomy	NA
	34	pelvic mass	18	-	resection of tumor	NA
Saeed AS [5]	27	pelvic mass	41	leiomyoma	ATH+BSO	NED, 1
Fukunaga M [14]	36	hypermenorrhea	4	-	ATH	NED, 96
	35	abdominal pain	18	-	ATH+BSO	NED, 144
Rocha AC [15]	38	abdominal pain	9	-	ATH+BS	NED, 18
Egashira H [6]	20s	abdominal distension	35	CDL	ATH+RSO+LS+OMT	NED, 4
Xu T [3]	37	pelvic mass	25	CDL	ATH+BSO	NED, 27

This highlights the difficulty of accurate diagnosis before and during surgery. Nevertheless, at least eight reported cases involved myomectomy or tumor reduction with the intent to preserve fertility, and at least one resulted in successful conception and delivery [8]. These reports suggest that conservative surgery is feasible in carefully selected patients [2,9]. Our case underscores both the potential and the limitations of this approach: although initial fertility-sparing resection was attempted, residual disease regrew rapidly and necessitated hysterectomy. Although CDL is generally regarded as non-recurrent, at least one case of recurrence has been documented, attributed to incomplete resection [10]. Our findings similarly emphasize that residual disease may enlarge quickly, highlighting the importance of complete removal when technically achievable. After definitive surgery, our patient has remained disease-free for two years.

The role of hormonal therapy in CDL remains insufficiently explored. Only two reports have described tumor reduction following GnRH analog treatment, suggesting possible responsiveness [8,16]. In conventional leiomyomas, PR expression usually exceeds ER expression, and GnRH agonists contribute to tumor shrinkage by downregulating both receptors. In contrast, in our case, GnRH antagonist therapy for four months after the initial surgery failed to induce tumor regression; instead, further growth occurred.

Before the second operation, ^18^F-FES PET was performed. This imaging modality noninvasively visualizes ER expression throughout the tumor and is considered useful for predicting hormonal therapy response in ER-positive lesions [17]. Because the tumor showed a poor response to hormonal therapy, ^18^F-FES PET was performed to assess the expression status of its hormone receptors. ^18^F-FES PET/MR showed ^18^F-FES uptake was observed in the endometrium, and no evidence of accumulation in the tumor. Although immunohistochemically ER-positive, the tumor showed low ^18^F-FES uptake. This discrepancy may reflect differences in ER subtypes detectable by ^18^F-FES or limitations in immunohistochemical evaluation [18,19]. Additionally, evidence from breast cancer studies suggests that prior exposure to anti-estrogen drugs can induce expression of nonfunctional ERs, resulting in diminished tracer uptake despite apparent ER positivity. ER expression may also vary among different tumor foci within the same patient or change following treatment [20].

In the present case, immunohistochemistry confirmed ER and PR positivity, yet ^18^F-FES uptake was low. This may indicate functionally impaired hormone receptors or other tumor-specific mechanisms, potentially explaining the lack of hormonal therapy response. Some CDL tumors, similar to ordinary leiomyomas, may exhibit hormone-independent growth, limiting the predictive value of ^18^F-FES PET in assessing responsiveness or guiding tumor size control. Consequently, to prevent recurrence or progression, complete surgical resection remains the cornerstone of management.

Taken together, these findings caution against overreliance on hormonal therapy in CDL, especially for residual or recurrent lesions. Although histologically benign, CDL can behave aggressively, with rapid enlargement, broad ligament extension, and regrowth after incomplete excision. Key management principles include: (1) Striving for complete resection whenever feasible to minimize recurrence. (2) Conducting careful intraoperative assessment to avoid overtreatment while maintaining oncologic safety. (3) Considering fertility-sparing surgery in reproductive-age women only if complete removal is achievable without injuring adjacent organs. (4) Recognizing that some CDLs, like conventional leiomyomas, may not respond to hormonal therapy.

## Conclusions

CDL is a histologically benign tumor that can exhibit aggressive behavior when incompletely excised. Our case demonstrates that incomplete excision may result in rapid regrowth, underscoring the importance of complete surgical removal whenever possible. Similarly, as in our case, hormonal therapy appears to have limited efficacy, possibly due to impaired or functionally inactive hormone receptors. Complete surgical resection remains essential to prevent recurrence, whereas fertility-sparing surgery should be considered only when complete removal is technically achievable. Future research should further evaluate the clinical utility of hormonal therapy and advanced imaging modalities such as ^18^F-FES PET to establish optimal management strategies for this challenging entity.
